# Distributed quasi-Bragg beam splitter in crossed atomic waveguides

**DOI:** 10.1038/s41598-017-04710-9

**Published:** 2017-07-06

**Authors:** V. Guarrera, R. Moore, A. Bunting, T. Vanderbruggen, Y. B. Ovchinnikov

**Affiliations:** 1 National Physical Laboratory, Teddington, TW11 0LW United Kingdom; 2 Midlands Ultracold Atom Research Centre, School of Physics and Astronomy, University of Birmingham, Edgbaston, Birmingham B15 2TT United Kingdom; 3 Department of Physics, University of Liverpool, Liverpool, L69 7ZE United Kingdom

## Abstract

We perform an experimental and theoretical study of a novel distributed quasi-Bragg splitter for cold atoms propagating in crossed optical waveguides. The atoms are guided by horizontal red-detuned laser beams which cross with an angle of roughly 90°. The lattice formed by the interference between the two waveguides is used as a quasi-Bragg splitter to *continuously* deflect the atomic flux from one waveguide into the other. In the limit of strong waveguide confinement and depending on the velocity of the cloud, three main regimes are observed corresponding (1) to the absence of reflection, (2) to partial reflection and (3) to full reflection into the second waveguide. In view of the application to atom interferometry, the condition to split the cloud into mainly two equally-populated fragments is only met in the highest velocity regime, where the fraction of reflected and transmitted atoms can be controlled by tuning the lattice height. A diagnostic of the momentum distribution shows that a quasi-Bragg splitter with the occupation of mainly two momentum states is achieved in this regime. This behaviour can be understood by considering the band structure associated with the potential in the crossing region and agrees with numerical simulations of the atomic dynamics.

## Introduction

Since the first realization of ultracold matter waves, the strong analogy with optics has boosted a number of seminal achievements and has driven novel technological progress^1–3^. A major goal in the field of atom optics is the realization of coherent atomic circuits. There is indeed a strong interest towards the potential technological application of these systems as guided atom interferometers^4^, and also as analogs of electronic circuits with enhanced control on the carrier particles, an emerging field known as Atomtronics^5, 6^. For all these applications, it is of primary importance to develop appropriate techniques to control the dynamics of the atoms in the waveguides, starting from the basic building block of any circuit and interferometer: the beam splitter. Coherent beam splitters have been realized for Bose-Einstein condensates (BECs) trapped in double-well potentials^7^, guided in linear optical waveguides^8^, and in Y-shaped waveguides with a small opening angle^9, 10^. However an integrated splitter able to continuously separate a guided atomic cloud and to spatially deflect it by a large and arbitrary angle has not been realized yet.

Here we consider a system where the atoms are confined in horizontal waveguides realized by means of red-detuned laser beams. The simple intersection of two such waveguides has shown not to lead to a good beam splitter as the atoms exit all the four available channels following chaotic dynamics^11^. In the domain of optical waveguides, a controlled reflection of the light is commonly achieved by employing distributed Bragg reflectors which serve as optical filters, couplers and optical (de)multiplexers. An analogous version for matter waves propagating in a single linear waveguide has been recently realized^12^.

**Figure 1:**
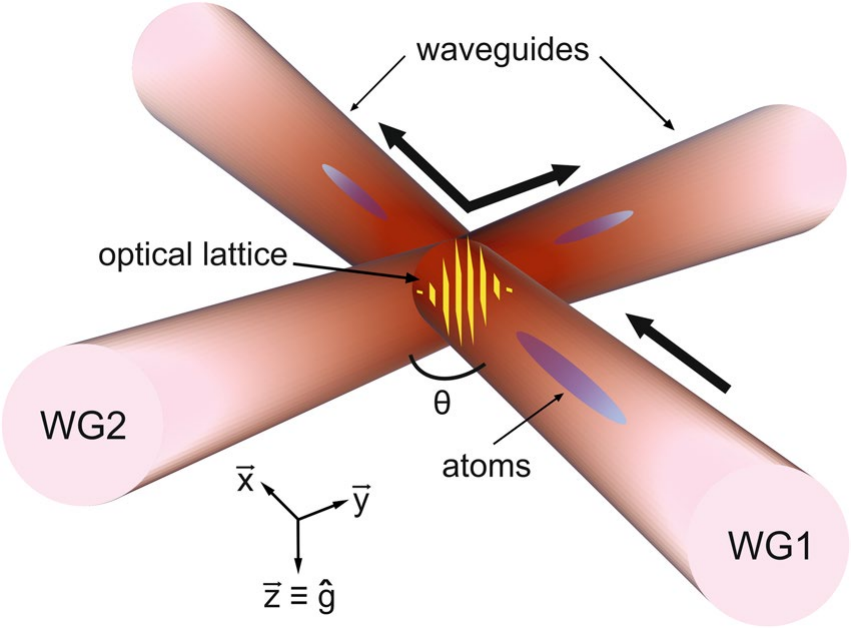
Schematics of the distributed quasi-Bragg reflector in crossing atomic waveguides. The two waveguides are realized by the same off-resonant laser and produce an inhomogeneous optical lattice in the crossing region where they interfere. We use this configuration as a controllable beam splitter to divide a BEC, which initially propagates along one linear waveguide (WG1), into two different fragments moving along each of the two waveguides (WG1 and WG2) which cross with a variable angle *θ*.

In this paper we study both theoretically and experimentally a novel all-optical splitter for propagating matter waves which combines for the first time these two features: a crossing guide configuration with a distributed quasi-Bragg reflector, where by *quasi-Bragg* we refer to an intermediate regime between the Bragg and the so-called *channeling* regime, where the diffraction pattern is still similar to the one generated by a Bragg diffraction^13, 14^. We propose to realize the periodic potential required for such a reflector by allowing the two crossing waveguides to interfere, thus generating an inhomogeneous optical lattice in the spatial region where they overlap, as shown in Fig. 1. The atoms initially propagating in one linear waveguide can thus be reflected by the lattice formed in the crossing region and can enter the second waveguide, which intercepts the first one at an angle *θ*. This minimal scheme thus allows a controlled reflection of the atoms into one specific direction of the crossing waveguide and could work for any angle *θ*, as the orientation of the lattice is naturally correlated with the alignment of the two waveguides. The proposed splitter could be used for Michelson as well as for Mach-Zehnder interferometry where a large deflection of the atomic beams can be exploited to design circuits with large enclosed areas for high-sensitivity measurements. In the latter case, the mirrors and the recombiner can be in principle realized with the same design of the splitter, provided four linear waveguides are properly intersected. We note that the recombination of two split clouds confined in waveguides can be done, without re-passing through the same path, either by bending the waveguides or by using straight waveguides and mirrors, similarly to optical interferometers. As bent optical dipole traps are not easy to realize^9^, our technique can provide a simpler solution for the generation of controlled atomic circuits with laser beams. Moreover, splitting and recombining the atoms in two momentum states provides an easier readout with respect to the Y-splitters, where the states to be read are external states of the radial trap realized by the waveguide. Our technique would ideally suit applications in optical waveguides realized by integrated or miniaturized optical elements. Atoms both in the BEC regime or in the thermal regime can be used, provided the momentum spread of the cloud is smaller than the recoil velocity which corresponds to a temperature much lower than 140 nK for our experimental parameters.

## Theoretical Description

We first theoretically study the proposed matterwave reflector in the simplified assumption of a two-dimensional (2D) system with no interparticle interactions, which is experimentally justified in the case of low atomic density. In the following we consider the motion of the atoms limited to a horizontal plane, as correction due to the gravitational sag is negligible in the considered range of parameters. The potential that results from the intersection between two identical Gaussian beams crossing near the two waists at an angle *θ* is $U(x,y)=-\frac{1}{2{\varepsilon }_{0}c}\alpha {|{E}_{0}|}^{2}I(x,y)$, where *α* is the atomic polarizability, *E*_0_ the amplitude of the light field and the normalized intensity *I*(*x*, *y*) in the assumption of ${w}_{0}\gg 2\pi /{k}_{R}$ is:

1 $$\begin{array}{rcl}I(x,y) & = & 2{e}^{-\frac{1}{{w}_{0}^{2}}[{(y\cos \theta -x\sin \theta )}^{2}+{y}^{2}]}\{\cosh \,[\frac{1}{{w}_{0}^{2}}[{(y\cos \theta -x\sin \theta )}^{2}-{y}^{2}]]\\  &  & +e\,\cos [{k}_{R}(x(\cos \,\theta -1)+y\,\sin \,\theta )]\}\end{array}$$

with *k*_*R*_ = 2*π*/*λ* the wave number of the light, *w*_0_ the beams’ waist and $0\le \varepsilon \le 1$ the interference contrast which depends on the relative polarization of the two beams and defines the lattice amplitude. In Eq. 1 the x-axis coincides with the axis of one of the waveguides, see also Fig. 1. To simplify the notation, we operate a change of coordinates consisting in a rotation of *θ*/2 and we work in the reference frame set by the lattice. The potential in the rotated basis {*x*′, *y*′} can be simply written as

2 $$U(x^{\prime} ,y^{\prime} )=-{U}_{0}(x^{\prime} ,y^{\prime} )[A(x^{\prime} ,y^{\prime} )+\varepsilon \,\cos ({q}_{R}(\theta )y^{\prime} )]$$

with the effective wavevector ${q}_{R}(\theta )=2\,\sin (\theta /2){k}_{R}=2\pi /d$ and *d* being the lattice spacing. In the intersection region, the potential *U*(*x*′, *y*′) is the sum of two terms: a trapping potential, $-{U}_{0}(x^{\prime} ,y^{\prime} )A(x^{\prime} ,y^{\prime} )$, and a quasi-periodic lattice with a gaussian envelope ${U}_{lat}=\varepsilon {U}_{0}(x^{\prime} ,y^{\prime} )$, being:

3 $$\begin{array}{rcl}{U}_{0}(x^{\prime} ,y^{\prime} ) & = & -2{U}_{t}{e}^{-\frac{1}{{w}_{0}^{2}}[(1-\cos \theta ){x}^{^{\prime} 2}+(1+\cos \theta ){y}^{^{\prime} 2}]}\\ A(x^{\prime} ,y^{\prime} ) & = & \cosh (2\,\sin \,\theta \frac{x^{\prime} y^{\prime} }{{w}_{0}^{2}}),\end{array}$$

where *U*_*t*_ is the trap depth of the single waveguide. Given ${w}_{0}\gg 2\pi /{k}_{R}$, we perform a local approximation assuming that the functions *U*_0_(*x*′, *y*′) and *A*(*x*′, *y*′) are slowly varying compared to cos (*q*_*R*_(*θ*)*y*′).

We thus study the local band gap structure produced by the potential $U(x^{\prime} ,y^{\prime} )$, using the method described in ref. 15. With the potential of Eq. 2, the stationary Schroedinger equation is a Mathieu equation and the problem of determining the band gaps is resolved by numerically calculating the imaginary part of the so-called Mathieu characteristic exponents *κ*. The solutions of the Mathieu equations have the form ${e}^{i\kappa x}f(x)$ and an imaginary value of *κ* indicates that the wavefunction is evanescent. For particle energies lying within a band gap, the transmission by the lattice is exponentially suppressed and reflection takes place. The inhomogeneity of the lattice amplitude has the effect of projecting in real space the band gap structure, giving rise to reflective barriers for the atoms called *spatial gaps*. The presence of an external confining potential, in addition, determines the spatial distribution of these gaps within the intersection region of the two crossing beams, where the lattice is formed. In particular, this is responsible for the lowest energy band gaps to appear at the external edge of the intersection region, the exact number of these *side* gaps depending on the depth of the trap.

**Figure 2:**
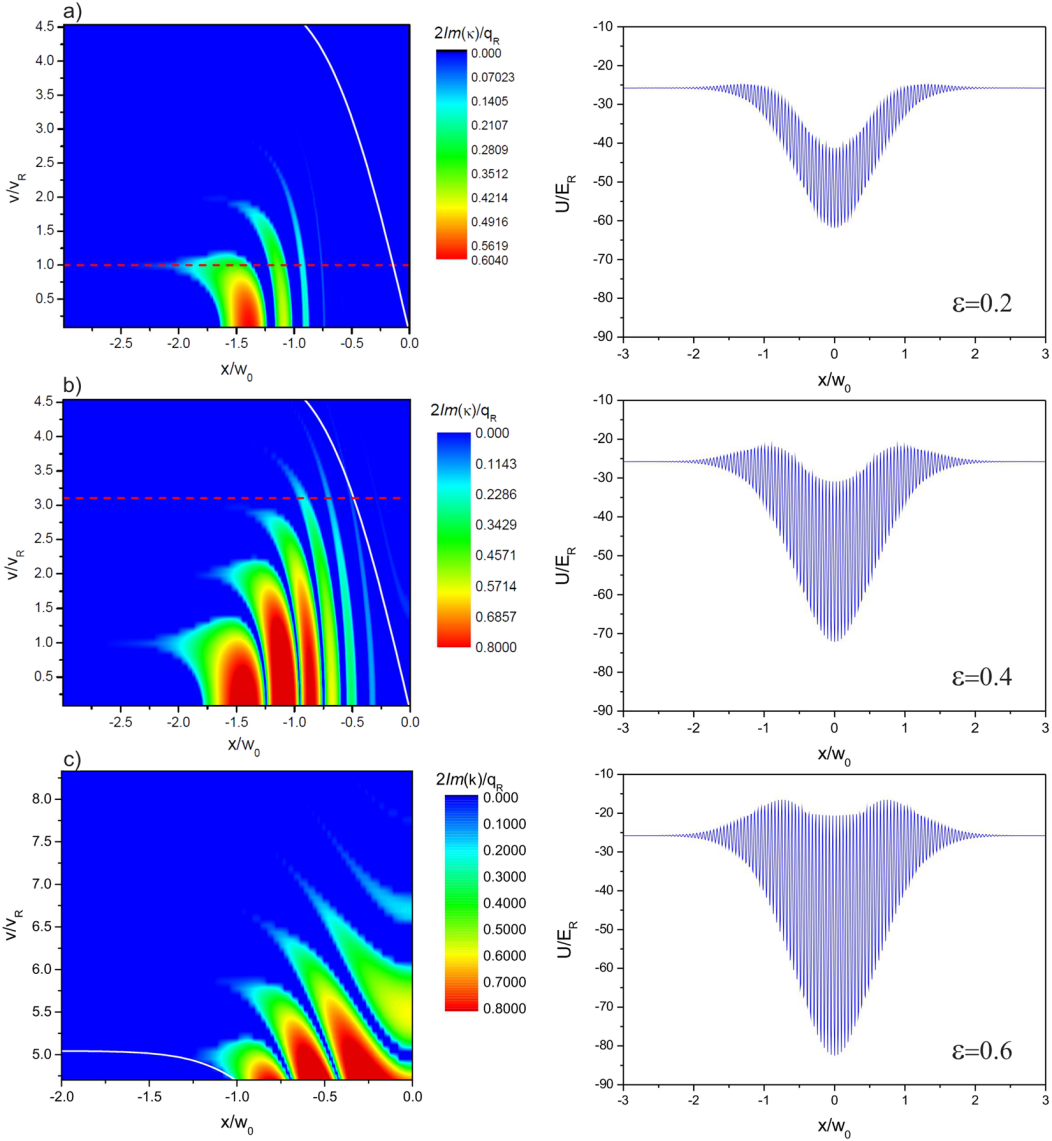
Imaginary part of the Mathieu characteristic exponent calculated along the *x* axis for different initial velocities of the atomic wavepacket and different lattice strengths. Atoms propagating towards the crossing region move from negative to positive values of *x*/*w*_0_ in this picture. The calculations refer to our experimental parameters with *λ* = 1064 nm, ${U}_{t}\simeq 25{E}_{R}$ and ${E}_{R}={\hslash }^{2}{k}_{R}^{2}/(2m)$. (**a**) For *ε* = 0.2 atoms with velocity v ≲ v_*R*_ (highlighted with a red dashed line) are most probably deflected by the lowest energy band opening at a distance ~1.4*w*_0_ from the center. (**b**) With *ε* = 0.4, higher order band gaps, opening progressively closer to the centre, deflect the atoms at velocities higher than v_*R*_. The solid white line in the graphs shows for each position *x* the initial velocity v = v_0_ at which the kinetic energy of the atoms satisfies the condition *E*_*kin*_(*x*, v_0_) = *U*_*t*_. When the atoms are reflected by the spatial gaps their kinetic energy is ideally transferred from the axis of one waveguide to the axis of the other. Only if ${E}_{kin}(x,v) > {U}_{t}$, or v > v_0_ for a position *x* where the reflection takes place, the atoms can leave the trap generated by the first waveguide along its radial direction and enter the second waveguide. The minimum velocity v_0_ at which a spatial gap intersects this condition, which for the case presented here is roughly equal to 3v_*R*_ (see red dashed line in the graph), provides a rough estimate of the onset of a regime where the atoms, once reflected by the gaps, can directly enter the second waveguide. (**c**) Zoom on the higher order band gaps opening in the centre of the crossing region, calculated for *ε* = 0.6. For each lattice height the corresponding potential $U(x,y=0)$ is shown in the right column of the figure. Note that the atoms are accelerated (and decelerated) when propagating across the intersection region due to the radial trap of the second waveguide with depth *U*_*t*_.

A full map of the spatial gaps in two dimensions is rather complex as it relies onto the details of the atomic dynamics in the crossing region. In the following we thus restrict this analysis to just one dimension by considering an atomic wavepacket propagating towards the intersection of the beams along the *x* axis as shown in Fig. 1 with an initial velocity v and total energy $E=\frac{1}{2}m\,{{\rm{v}}}^{2}+U{|}_{(x=-\delta ,y=\mathrm{0)}}$, where $\delta \gg {w}_{0}$ is the distance between the position where the atoms are released and the center of the crossing. The angle formed by the two waveguides *θ* = 90° has been also chosen, in the first instance, to avoid the coupling of the axial and radial degrees of freedom in the waveguides which takes place when an atomic cloud propagates in the intersection region of two non-interfering crossing beams, as shown in ref. 11. It is also important to notice that a large trap depth with ${U}_{t}\gg {E}_{R}=\frac{{\hslash }^{2}{k}_{R}^{2}}{2m}$ is necessary to hold the atoms against gravity during their motion in the waveguides for ${w}_{0}\gg \lambda$. For a given lattice amplitude *ε*, we plot in Fig. 2 the imaginary part of *κ* at the crossing region versus the velocity of the incoming particle to obtain a 1D map of the spatial regions where the atoms can undergo a reflection. For a given trap depth *U*_*t*_, the location of these regions depends on the atom velocity and on the lattice amplitude. The atoms with an initial velocity lower than the recoil velocity *v*_*R*_ are mainly deflected by the first spatial band gap opening at a distance larger than the beam waist *w*_0_ from the center of the intersection, starting from very low lattice amplitudes Fig. 2a,b. Increasing the initial velocity, the effect of the low lying energy band gaps progressively fades and only higher order band gaps opening closer to the centre of the crossing region count, as shown in Fig. 2b. Obviously, a lower value of *κ* and a smaller spatial width of the gap, which means a reduced probability of reflection, are associated with the higher order band gaps for a certain lattice strength. However, by tuning these parameters, it should be possible to control both the amount of reflected atoms and the position where reflection takes place, including the center of the crossing region as shown in Fig. 2c. We note that at the external edge of the crossing region, where the lattice amplitude is arbitrarily low, the usual Bragg condition holds with reflection taking place for velocities which are integer multiples of the recoil velocity v = *N* × v_*R*_ = *N* × *ħk*_*R*_/*m*, these values are also independent of the crossing angle *θ*.

In order to get a more analytical insight on the shape of the spatial gaps, it is instructive to extend the perturbative approach of ref. 15, and calculate the energy borders of the spatial gaps in the limit of low lattice depth. For a higher order band gap, the second order for example, by using a three-modes approximation we obtain:

4 $$\begin{array}{rcl}{E}_{+}^{(n=2)}(x^{\prime} ,y^{\prime} ) & = & {E}_{R}\,(\frac{1}{2}-A(x^{\prime} ,y^{\prime} ){s}_{0}(x^{\prime} ,y^{\prime} )+\frac{1}{2}\sqrt{1+2{\varepsilon }^{2}{s}_{0}^{2}})\\ {E}_{-}^{(n=2)}(x^{\prime} ,y^{\prime} ) & = & {E}_{R}\,(1-A(x^{\prime} ,y^{\prime} ){s}_{0}(x^{\prime} ,y^{\prime} )),\end{array}$$

where ${s}_{0}(x^{\prime} ,y^{\prime} )={U}_{0}(x^{\prime} ,y^{\prime} )/{E}_{R}$. It can be seen that the shape of the spatial gap depends on the envelope of the inhomogeneous lattice $\varepsilon {s}_{0}(x^{\prime} ,y^{\prime} )$, and on the trap $A(x^{\prime} ,y^{\prime} ){s}_{0}(x^{\prime} ,y^{\prime} )$, which acts as an energy offset shifting the gap at lower energies as it approaches the center of the crossing region. From the thickness of the gap ${E}_{+}^{(n)}(x^{\prime} ,y^{\prime} )-{E}_{-}^{(n)}(x^{\prime} ,y^{\prime} )$, which depends on the amplitude of the lattice, we can derive an upper limit for the spread of the initial velocity of the atomic cloud. This can be estimated on the order of few tenths v_*R*_ for typical parameters of our splitter. The velocity spread fundamentally limits the efficiency of a Bragg splitter^16^. In our case, due to the spatial inhomogeneity, the distribution of the initial velocities also determines the extension of the region where the atoms are reflected. A velocity spread much smaller than the recoil velocity v_*R*_ is thus necessary for mode-matching of the reflected cloud to the radial trap of the second waveguide. For our experimental parameters this implies that the temperature of the atomic cloud should be much lower than 140 nK, which indicates that a Bose-Einstein condensate is the ideal atomic source to be used with the splitter.

Finally, we complement the theoretical description provided by the Mathieu equation approach with the simulation of the atomic dynamics. This is done by numerically solving the time-dependent Schroedinger equation for a BEC in 2D with the split-step Fourier method. The calculations are performed for ^87^Rb atoms, external potential equal to *U*(*x*, *y*) and in absence of interparticle interactions. The results obtained with the two different approaches are consistent. While the spatial band gaps calculated within the Mathieu equation approach provide a general understanding of the interaction of the atoms with the inhomogeneous lattice, the solution of the time-dependent Schroedinger equation reveals all the details of the complex two-dimensional dynamics that the atoms undergo in the crossing region.

## Experimental Results

We prepare a spinor BEC of 7 × 10^4^ Rb atoms and we release it in a linear waveguide (WG1) by rapidly switching off the beams of the crossed dipole trap where the BEC is initially produced. The atoms can thus propagate in the harmonic trap formed by WG1, which has an axial frequency *ω*_*ax*_ = 2*π* × 2.4 Hz and a radial frequency *ω*_*rad*_ = 2*π* × 190 Hz. A second similar waveguide (WG2) is aligned orthogonally to WG1 at a distance *δ* from the position where the BEC is generated and in such a way that the two waveguides intersect at their minimum waist position, see Methods for more details on the experimental apparatus. Both the waveguides are created by linearly polarized laser beams with waist *w*_0_ ≃ 20 *μ*m and wavelength *λ* = 1064 nm. Each waveguide produces at the intersection region a trapping potential *U*_*t*_ ~ 25*E*_*R*_. Once the atoms are released in WG1 they naturally move towards the minimum of this trap, which coincides with the position of the minimum waist, thus acquiring a maximum velocity v ≃ *ω*_*ax*_ *δ* immediately before the intersection between WG1 and WG2. The measured velocity spread *δ*v = ±0.2 v_*R*_, where v_*R*_ = 4.3 mm/s is the recoil velocity, is mainly due to the interaction energy of the atoms converting into kinetic energy as they are released from the initial trap. Being derived from the same laser, WG1 and WG2 can interfere at their crossing, thus forming an optical lattice whose height can be set by tuning the relative polarization between the two beams. By changing the distance *δ* we can control the velocity of the atomic cloud center of mass at the lattice position, while maintaining the same atomic density after a fixed expansion time of 100 ms, avoiding effects due to the interactions. The maximum velocity we can achieve in this way is roughly 4.7 v_*R*_ corresponding to a distance *δ* ~ 1 mm equal to the Rayleigh length of the WG1 Gaussian beam.

**Figure 3:**
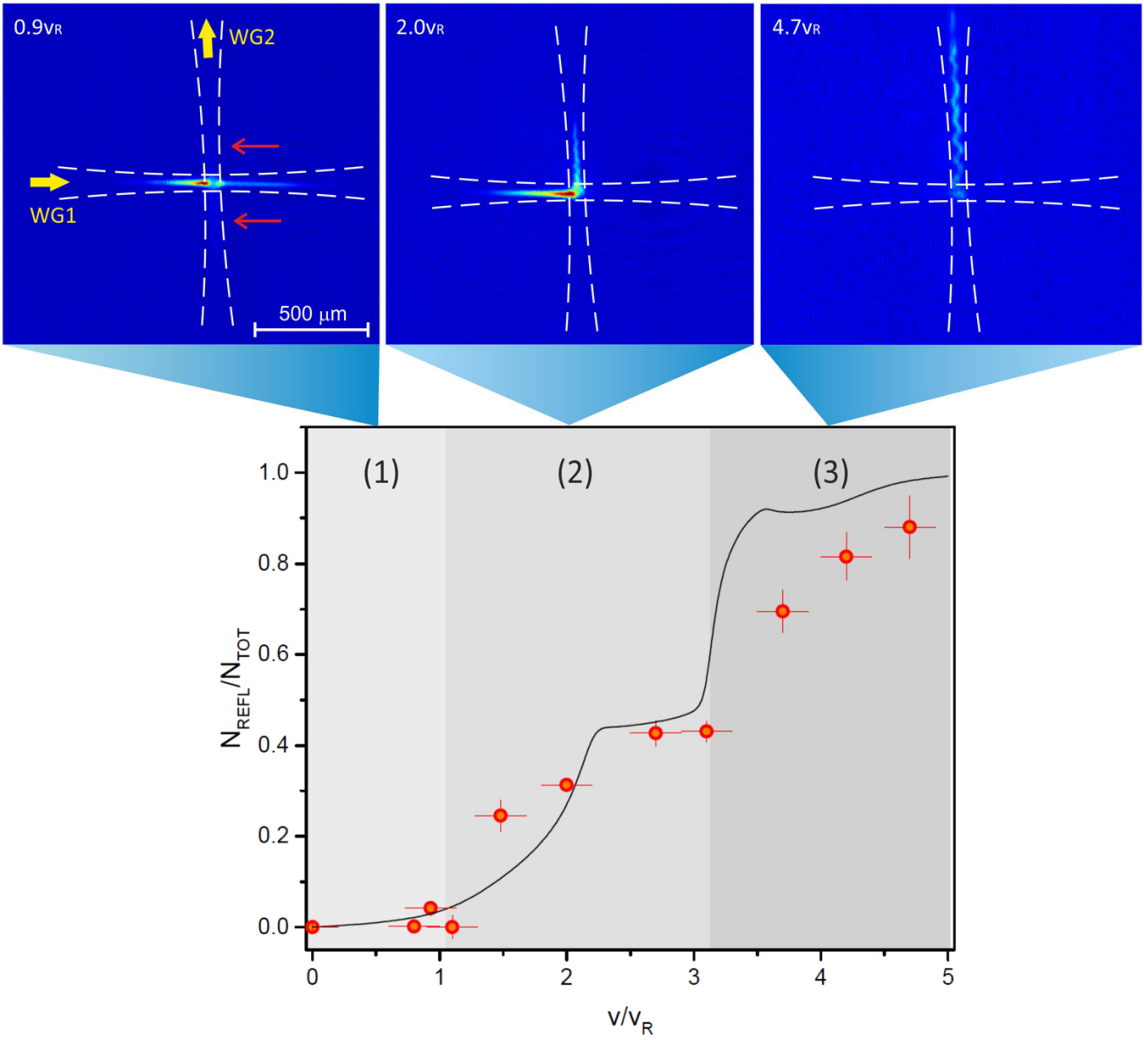
The maximum fraction of atoms reflected into WG2 obtained by optimizing *ε*, here plotted as a function of the initial velocity of the BEC. Three regimes of propagation are observed experimentally: (1) for low velocity v ≲ v_*R*_ the atoms stop at the edge of the crossing region and only a few atoms enter WG2 propagating in both its two directions (this weak signal in WG2 is highlighted by the red arrows), (2) for higher velocities v_*R*_ < v ≲ 3v_*R*_ the atoms start to be reflected into a single port of WG2, (3) when v > 3v_*R*_ the atoms are almost fully reflected into WG2. The atomic clouds are imaged after passing through the intersection region, for different lattice amplitudes, with 2 ms time-of-flight. White dashed lines are added to the images of the atoms to show the orientation of the two waveguides. The black solid line in the graph refers to the numerical solution of the time-dependent Schroedinger equation in 2D, which shows a good agreement with the experimental data.

**Figure 4:**
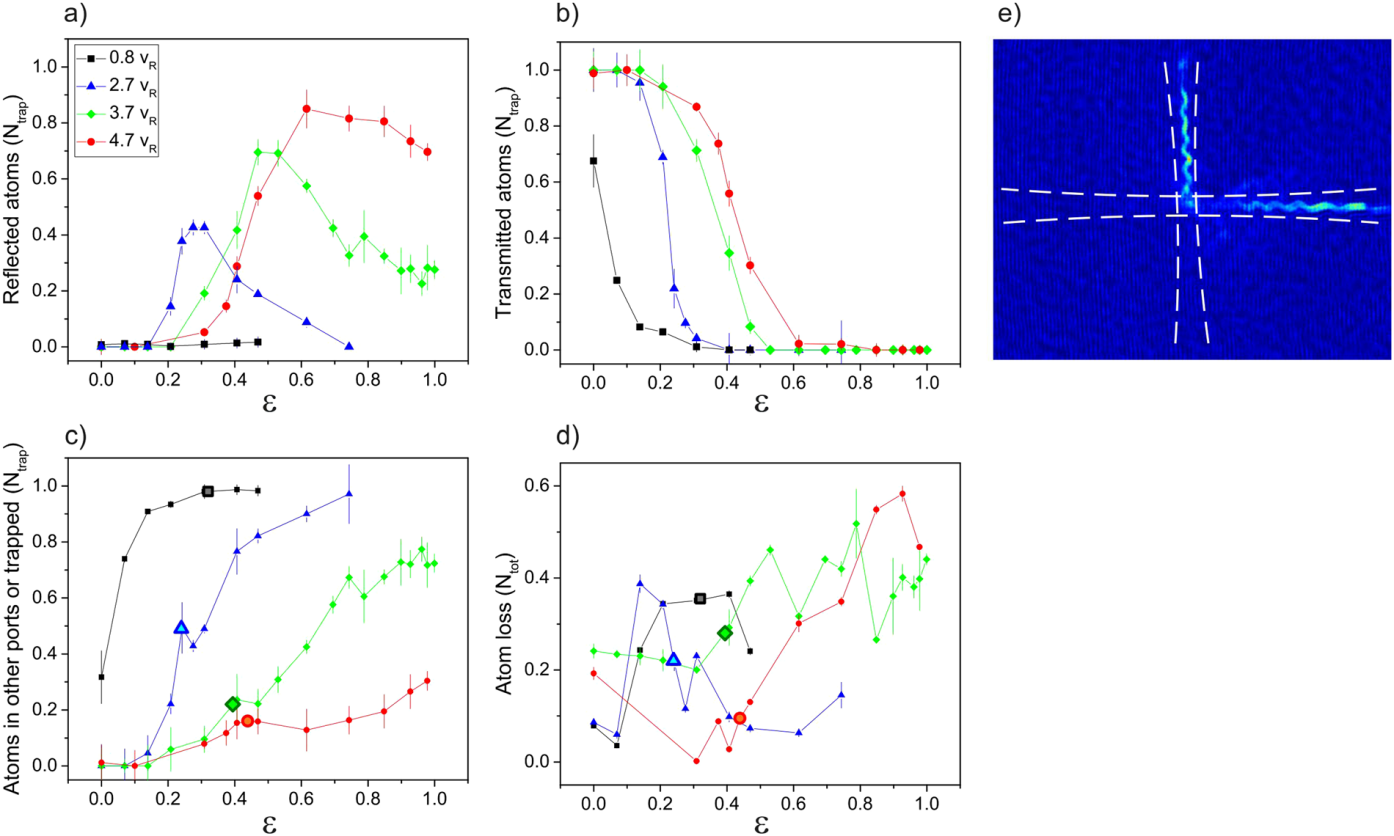
Number of atoms which are (**a**) reflected into WG2 along a single port, (**b**) transmitted in WG1, and (**c**) lost in the other two ports or trapped in the crossing region, as a function of the lattice height *ε* for four different propagation velocities. The measurements are taken roughly 20 ms after the atoms first arrived at the crossing and are normalized by the total atom number *in the trap*. Information on the losses from the trap following the passage through the crossing is reported in (**d**). The relative difference in the atom number measured before and after the interaction with the lattice is shown. In (**c**,**d**) we have highlighted with larger symbols the values which correspond to an equal splitting between the two exit ports in WG1 and WG2. Solid lines are guides for the eyes. (**e**) For v ~5v_*R*_ and larger, the atomic cloud can be split into two equally populated fragments propagating along WG1 and WG2 with best efficiency around 80% (comprehensive of the losses from the trap). Note that, by tuning the lattice height in this regime, the splitter can be also used as a mirror with best efficiency larger than 60% (comprehensive of the losses from the trap).

We study the dynamics of the atomic wavepacket when passing through the crossing region with different velocities and for different lattice heights. In our measurements we can identify three main regimes, in the limit of strong confinement ${U}_{t}\gg {E}_{R}$. For low velocities v ≤ v_1_ ≃ v_*R*_, the atoms are blocked at the external edge of the intersection region for very low lattice amplitudes and we do not observe any macroscopic reflection into WG2, see Figs 3 and 4. This behaviour can be explained recalling the band gaps structure: the atoms are reflected from the first band gap at a distance larger than *w*_0_ from the center of the crossing, the precise position depending on the initial velocity of the wavepacket. At this point, they have not acquired sufficient kinetic energy to leave WG1 and they remain confined in the combined trap formed by the lattice and the trapping potential of the waveguides. Only very few atoms, see Fig. 3-(1) for example, can enter WG2 in both directions for intermediate lattice strengths, by redistributing isotropically after passing some time inside the crossing region. No clear effect related to the lattice orientation is visible in the propagation of the atoms in this case and the presence of the trapping potential, due to the crossing of the two waveguides, has a major role in the resulting complex dynamics. This behaviour, which is also confirmed by the numerical solution of the 2D time-dependent Schroedinger equation, could signal the onset of a chaotic regime of propagation similar to the one described in ref. 11.

For intermediate velocity v_1_ < v ≤ v_2_ ≃ 3v_*R*_, we observe a substantial reflection into WG2. Indeed, while a fraction of the atoms are still reflected in both directions of WG2 for intermediate lattice amplitudes, the cloud is more consistently deflected into one single exit port of WG2 for increasing lattice strengths. At low lattice strength, the atoms are broadly diffused from the center of the intersection region, whereas for higher lattice height they are reflected out of the center of WG2, and acquire an oscillation along the radial direction when later propagating in WG2 see Fig. 3-(2). This signals that the atoms with sufficiently high velocity can pass unaffected by the first energy gap and are reflected by higher order gaps. In this case, these are the second, third, and fourth order gaps, which open closer to the crossing center at a distance ~*w*_0_, as derived from the solution of the Mathieu equation. Even if this results in a higher probability of reflection into WG2, at this distance however the atoms cannot yet directly enter WG2 once reflected by the band gaps, see Fig. 2b. Motivated by the good agreement between the experimental measurements and the results of the numerical simulations shown in Fig. 3, we can rely on the latter for understanding the mechanism responsible for guiding the atoms into WG2 after reflection by the spatial gaps. We observe a progressive change from a situation where these dynamics are mainly driven by the boundaries of the waveguides’ trapping potential at low velocities, to a *cascade* of multiple reflections by several spatial gaps opening in the *xy*-plane for increasing atomic velocities. Additionally, the fraction of atoms reflected into WG2 (in a single port) is always below 50%, see Figs 3 and 4a. Indeed, for increasing lattice depths the atoms are blocked at the edge of the crossing region in WG1 as shown in Fig. 4c, see also Methods for a more detailed analysis of the atom number in the different exit ports and in the crossing region. Note that quantum reflection by a *single* lattice site, estimated analytically by using a similar barrier shape^17^, occurs only for relatively high lattice amplitudes in this range of velocities, see Methods. Neither the observed reflection into WG2 or the blocking of the atoms at the edge of the crossing region can be explained with reflection by a *single* barrier. They are thus due to the periodic potential and, more specifically, to the action of spatial gaps of different order.

Finally, when increasing the velocity above v_2_ an increasingly larger fraction of the atoms are reflected into WG2, to the point where an almost complete reflection can be achieved (85% of the atoms in the trap for the maximum velocity reported in Fig. 3). The higher order band gaps are now responsible for the reflection of the atoms at a distance smaller than *w*_0_ from the center of the crossing. In this regime, atoms can exit WG1 to enter WG2 both directly or after a few subsequent reflections, depending on their initial velocity and lattice height. In the limit of large lattice amplitudes, the reflection probability drops again, Fig. 4a–c, likely due to the onset of single-barrier quantum reflection, which is independent of the band gap spatial distribution and shows a reflection peak around *x* ~ *w*_0_, see Methods. We note that these different regimes of propagation generally appear in the limit ${U}_{t}\gg {E}_{R}$ when the atoms naturally cross several spatial bands due to the strong trapping potential and only v_2_ depends on the details of the specific realization. Interestingly enough in this regime of high velocity we can fully control the ratio of atoms reflected and transmitted by the lattice by adjusting the lattice height *ε* and we can also split the atoms into two equally populated fragments propagating in WG1 and WG2 respectively, Fig. 4e. As anticipated above, this feature is of great interest in the perspective of realizing an all-optical guided atom interferometer. For this purpose it is also of primary importance to perform an accurate diagnostic on the status of the BEC after the splitting. Not only should the splitter be coherent but it should also not generate excitations which limit the coherence time, and thus the sensitivity of the interferometer, or which smear out the contrast of the interferometric fringes by populating too many modes of the confining trap^18^. For the tested range of initial velocities, we have generally observed a fragmentation of the cloud into a few pieces after the splitting, accompanied by excitations: whereas the transmitted cloud shows a clear oscillation of the centre-of-mass in the radial direction of the waveguide, the reflected one exhibits a more complex excitation, as also confirmed by the results of the numerical simulations. We have detected atom losses from the trap both in the splitter configuration, 20% on average for the velocity range 3v_*R*_ − 5v_*R*_, and in the mirror configuration, 30% roughly, see also Fig. 4d.

To better explore this regime for application to atom interferometry, we have acquired a second set of measurements focussing on reaching even higher velocities of propagation. In this case, the BEC is spin-purified during the evaporation process and the atoms occupy the state |*F* = 1, *m*_*F*_ = −1〉 with a purity larger than 95%. Wavepacket velocities up to 23v_*R*_ are obtained by applying a magnetic field gradient along the axial direction of WG1, before the atoms arrive at the crossing region between the two waveguides. Experimental results show a reduced cloud fragmentation and excitation when the splitting is obtained for velocities roughly higher than 5v_*R*_. This is also reflected in the average atom losses which decrease to 7%. This is compatible with the fact that the atoms can be reflected only by a reduced number of band gaps which open near or at the center of the crossing region for particle energies larger than the trap depth, as shown in Fig. 2c. The closer the reflection takes place with respect to the axis of WG2, the smaller is the amplitude of the radial oscillation that the cloud undergoes when propagating into WG2. Additionally the full two-dimensional dynamics is greatly simplified as the atoms can be directly reflected into WG2 without undergoing several subsequent partial reflections. For the transmitted cloud, the observed radial oscillation of its centre of mass does not appreciably change with the initial velocity in the range that we have considered. This can be explained by the fact that for strong lattice amplitudes ${U}_{lat}\ge \frac{1}{2}m{{\rm{v}}}^{2}$, the atoms start shifting in direction of the channels of the optical lattice near the center of the splitter^14^.

**Figure 5:**
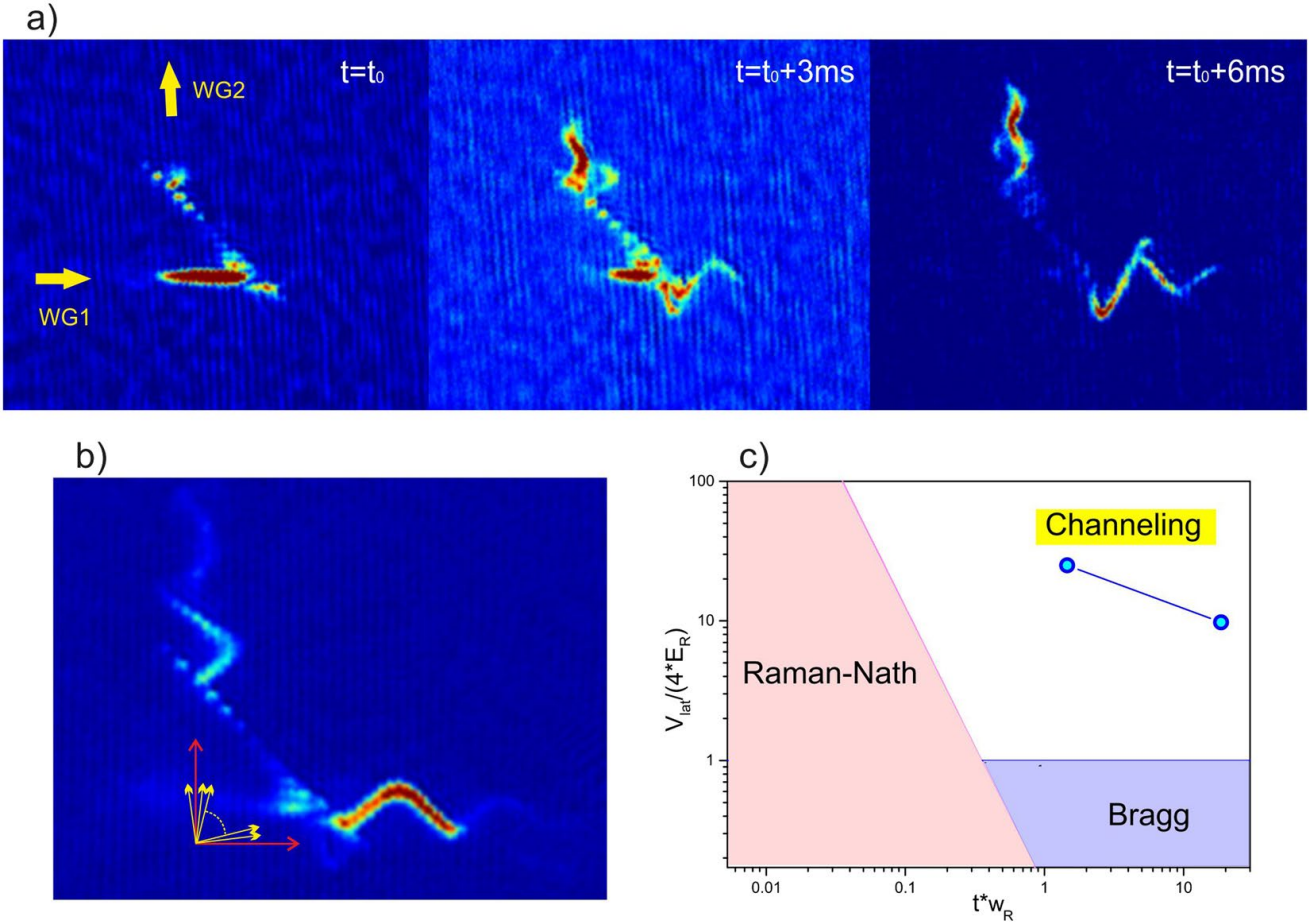
(**a**) Atomic clouds imaged at different times when passing through the quasi-Bragg splitter and after 5 ms time-of-flight. The BEC velocity before entering the reflector is (7.0 ± 0.2)v_*R*_, the height of the lattice is $\varepsilon =0.92$, and *t*_0_ = 40 ms is the time the BEC takes to move from its initial position to the splitter. The population of several quasi-momentum peaks is visible around **q**_1,2_ signalling the presence of multi-photon processes in the interaction with the strong lattice. The quasi-momentum distribution also provides an effective ruler for the understanding of the momentum distribution of the atoms when leaving the splitter. For the measurements shown here, for example, one can see that the reflected and transmitted atoms occupy a few different momentum states after the splitting. (**b**) For high enough initial velocities, two main momentum states can be occupied in the exit ports of the splitter. The picture shows a measurement taken for v = (8.1 ± 0.2)v_*R*_ at *t* = 40 ms with $\varepsilon =1.0$. (**c**) Our beam splitter lies between the Bragg and the “channeling” regime for the velocity range that we have investigated, i.e. 3v_*R*_ ≤ v ≤ 8v_*R*_. However, thanks to the smooth Gaussian envelope of the lattice, the diffraction pattern produced by a single spatial gap is very similar to that typical of a Bragg regime. We refer to this regime as *quasi-Bragg*.

Further comprehension of these dynamics are obtained by rapidly switching off all the confining potentials and by analysing the atomic cloud after a few ms time-of-flight expansion. Interestingly, when observing the atoms during the splitting phase we can clearly resolve several quasi-momentum peaks. These peaks reveal the several multi-photon processes that the atoms undergo in the interaction with the light grating before their direction of propagation is deflected by an angle close to 90°. The velocity of propagation and the envelope of the inhomogeneous lattice are naturally setting the thickness of the light grating. For our experimental parameters the interaction time between the atoms and the optical lattice is typically long $(t\frac{{E}_{R}}{\hslash } > 1)$ and the reflection of the atoms takes place at strong potentials (${U}_{lat}\gg {E}_{R}$), which characterize our splitter to lie between the Bragg and the *channeling* regime using the nomenclature of refs 13, 14, see Fig. 5c. The diffraction grating also provides a ruler for the diagnostics of the momentum distribution of the cloud after the splitting. In Fig. 5 we report an example of the time evolution of the quasi-momentum peaks. We first observe that few quasi-momentum peaks are populated around ${{\bf{q}}}_{1}=(q,0)$ and ${{\bf{q}}}_{2}=(0,q)$ where *q* corresponds to the momentum of the atoms before the reflection. Adjacent peaks are separated by ${\rm{\Delta }}{\bf{q}}=\pm ({q}_{R}(\theta )\cos (\frac{\pi -\theta }{2}),{q}_{R}(\theta )\sin (\frac{\pi -\theta }{2}))$. The number of occupied peaks increases in time as they cover the whole range between **q**_1_ and **q**_2_, signalling that several multi-photon processes, instead of two-photon like processes, have taken place in-between the two extreme quasi-momentum states. Observing the dynamics at a later time, it is possible to identify which momentum the atoms have acquired when leaving the splitter. The number of momentum states occupied can be controlled by changing the initial velocity of the atoms. Despite the complexity of the dynamics undergone by the atoms in the splitter, for an initial velocity as high as 8v_*R*_, one single momentum state can be occupied in each exit port, see Fig. 5b. This confirms that the splitting is due to diffraction by the off-resonant lattice in a coherent process, just like in a standard Bragg diffraction. Moreover it shows that the atoms are not reflected by several band gaps or scattered by the walls of the confining potential. Indeed all these dynamics, which can lead to loss of coherence, appear with the occupation of other momentum states out of the splitter. The occupation of mainly two momentum states corresponds to the observed removal of the cloud fragmentation and to the realization of a *quasi-Bragg* splitter with $16\hslash {{\bf{q}}}_{R}$ momentum transfer. Note that a residual oscillation is present in the transverse direction of the waveguide both for the reflected and transmitted clouds which is compatible with deflection of the center of mass motion due to the onset of channeling. Finally, we point out that the splitter does not substantially improve for velocities larger than 8v_*R*_, which require larger lattice amplitudes and thus larger potential depths for the cloud splitting to be achieved.

## Discussion and Conclusions

**Figure 6:**
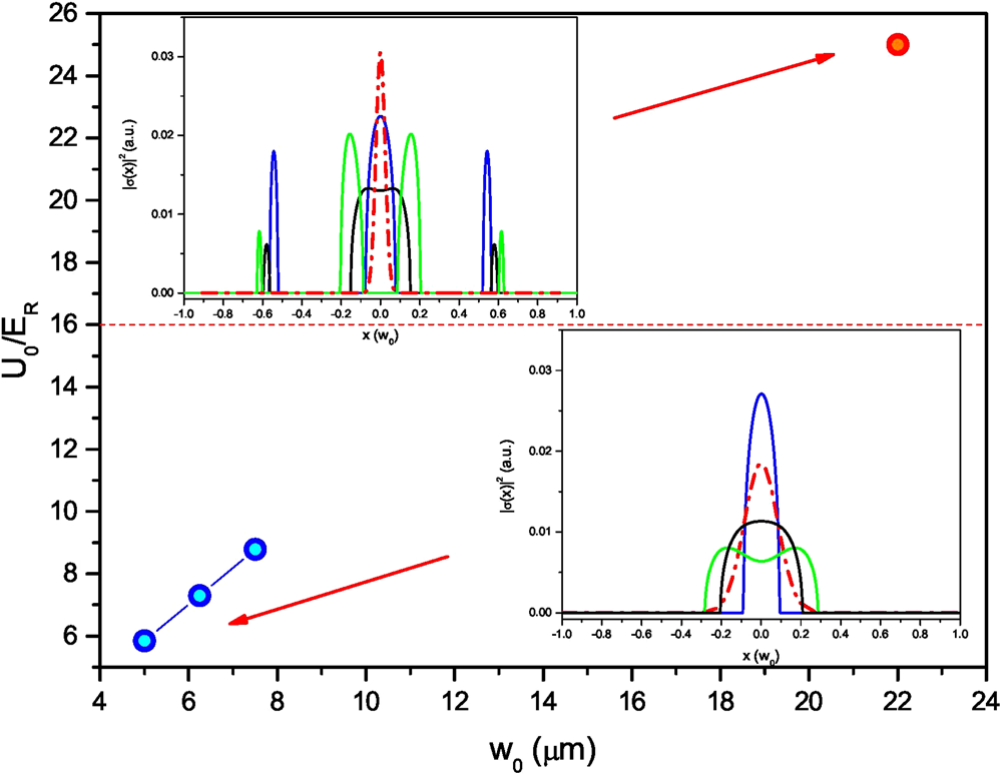
The main parameters of the quasi-Bragg splitter (trap depth and beam waist) are shown for different realizations numerically optimized to minimize the generation of radial excitations when dividing the cloud into two equal parts. Solving the relative Mathieu equations, we observe a crossover from a single isolated spatial gap appearing at the centre of the crossing region (*w*_0_ ≤ 8 *μ*m, shown with blue dots in the graph) to two distinct gaps (red dot refers to our experimental realization). The dashed line is a simple estimate of the upper boundary for an isolated band gap to appear, calculated in the limit ${U}_{t}\gg {E}_{R}$. In the insets we report the section of the spatial band gaps ${|\sigma (x)|}^{2}$ for the optimized splitters with *w*_0_ = 5 *μ*m and *w*_0_ = 22 *μ*m. For *w*_0_ = 5 *μ*m the spatial gap has been calculated for three different initial velocities v = (3.94, 4.04, 4.14)v_*R*_ with spread *δ*v = 0.2v_*R*_ (solid lines in the inset) and for maximum lattice amplitude 22*E*_*R*_. For *w*_0_ = 22 *μ*m, the calculation has been done for *ε* = v = (7.90, 8.00, 8.10)v_*R*_ with maximum lattice amplitude of 100*E*_*R*_. The dash-dotted lines show, for comparison, the density distribution of the radial ground state in WG2.

We have presented the realization of the first distributed quasi-Bragg splitter for ultracold atoms confined in crossed optical waveguides which is able to split the atoms with a large angle of roughly 90° and overall efficiency of 80%. A splitter for atom interferometry should ideally lead to the occupation of two momentum states with no further excitation produced. For a pulsed quasi-Bragg splitter with a Gaussian envelope in time, the population in the scattering orders other than those allowed by the Bragg condition can be very small when the pulse has a smooth envelope function even out of the Bragg regime^13, 14^. In this case the typical timescales required to guarantee the occupation of the two target momentum states are consistently shorter than the ones imposed by the adiabaticity criterion. In our splitter, the atomic dynamics determines the interaction time with the inhomogeneous lattice, which is equivalent to having a smooth pulse with timescales which are almost two orders of magnitude slower than the ones compatible with negligible losses^13^. We have also pointed out that, to guarantee the occupation of mainly two momentum states, the atoms should ideally interact with one single spatial gap. Additionally, to avoid radial excitations of the split cloud, the atoms should be reflected in the centre of the crossing region. These conditions can be better met by working in a regime of high velocity, for the experimental parameters which we have considered here v ≳8v_*R*_, or they can be satisfied at lower velocity by reducing the depth of the confining potential due to the waveguides. Considering the gravity sag and without reducing the radial confinement of the trap (see discussion below), the second option turns out to be possible only by reducing the radial size of the waveguides. In Fig. 6, we have reported the spatial sections of the lowest-energy gaps providing the best condition for the beam splitting. It can be seen that lower energy band gaps can be used for the splitter when smaller waveguides are employed (blue points in the graph). In this case, a single spatial gap is found in the center of the crossing with less sensitivity on the initial velocity spread (see insets of Fig. 6) due to the reduced trap depth. This configuration appears more promising than the best experimental realization presented in this work (red point in the graph). However, even for those parameters and despite the good spatial overlap of the band gaps with the radial ground state of WG2 (on average larger than 75% for a velocity spread of 0.2v_*R*_, with beam waist of 5 *μ*m), we have numerically found a non-zero probability of occupation of several excited states. The reflection process is indeed taking place on time-scales which are comparable to those set by the radial frequency of the waveguides and the transfer of the atoms from WG1 to WG2 results in a non-adiabatic process. For reducing these effects, a possibility consists in decreasing the angle of intersection between the waveguides ($\theta \le {3}^{\circ }$ for the experimental realization presented here)^10^. Another solution consists in using waveguides of even smaller size than the ones reported in Fig. 6. We note that the conclusions drawn so far are only valid in the limit ${w}_{0}\gg \lambda /2$, whereas for smaller beam waists the physical picture changes: matterwave reflection is not following the cumulative action of an extended lattice potential but it is rather a single-barrier induced effect. We have numerically verified that single radial modes can be addressed in the exit ports of the splitter for waveguide beam waists smaller than 5 *μ*m (and *λ* = 1064 nm) with a coherence preserving quasi-adiabatic approach. Even though this may be difficult to achieve in free space experiments where the waveguides are realized by single Gaussian beams, it is experimentally feasible when state-of-the-art all optical waveguides in planar chip configurations are employed^19^.

## Methods

### Experimental setup

**Figure 7:**
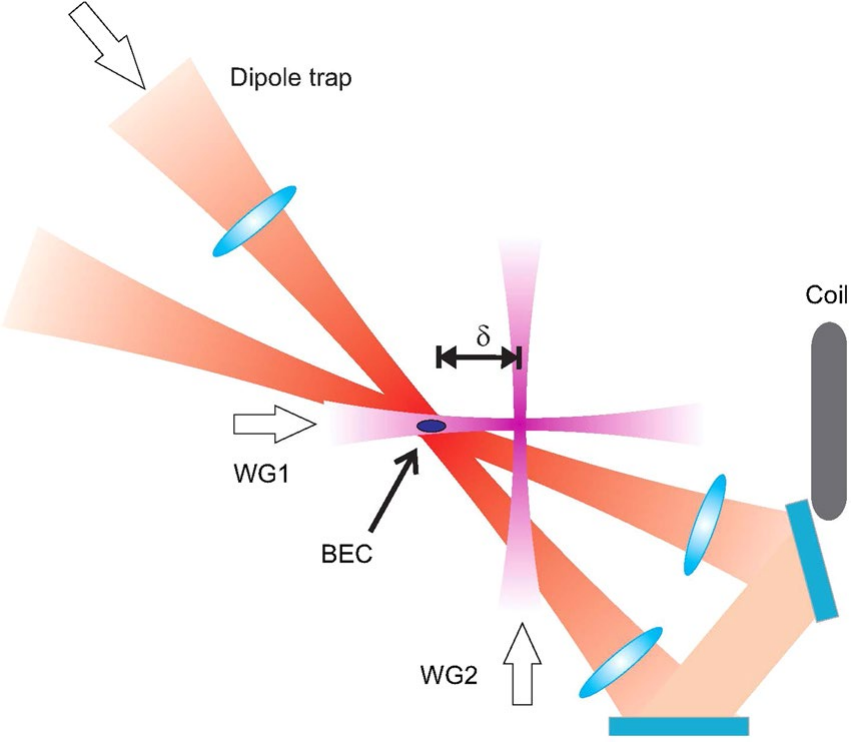
Schematics of the experimental apparatus.

In our experiment we produce ^87^Rb Bose-Einstein condensates in an all optical way. We use a crossed dipole trap realized by a recirculating single laser beam with 1064 nm wavelength, 18 W initial power and 65 *μ*m waist at the position of the atoms. After a first passage through the cell, the beam is reflected back with orthogonal polarization and refocussed to the atomic cloud in such a way to form an angle of 25° with respect to the incoming direction of propagation. In order to evaporatively cool the sample, the power of the trap beam is decreased with an exponential ramp of 8 s duration. For the measurements presented here, we prepare the BEC in the combined trap realized by the superposition of the crossed dipole trap and a waveguide (WG1) which acts as a dimple. This allows us to prepare the initial condensate in the radial ground state of WG1. The waveguide is created by means of a linearly polarized laser beam with *λ* = 1064 nm, power 15.5 mW and minimum waist of 22 *μ*m. To avoid cross interference between the WG1 and the dipole trap, the first beam is shifted in frequency by 80 MHz with respect to the second one. The WG1 beam is focussed at a distance *δ* from the center of the dipole trap where the BEC is formed. Another almost identical waveguide (WG2), with waist 24 *μ*m, power 17 mW and same frequency as WG1, is aligned with focal position coinciding with WG1 and forming with it an angle of roughly 90°, see Fig. 7. The amplitude of the waveguide potential is set by the minimum power necessary for holding the atoms against gravity at a distance of 1 mm from the position of the focus, where the BEC is initially prepared.

### Dynamics in the different regimes

**Figure 8:**
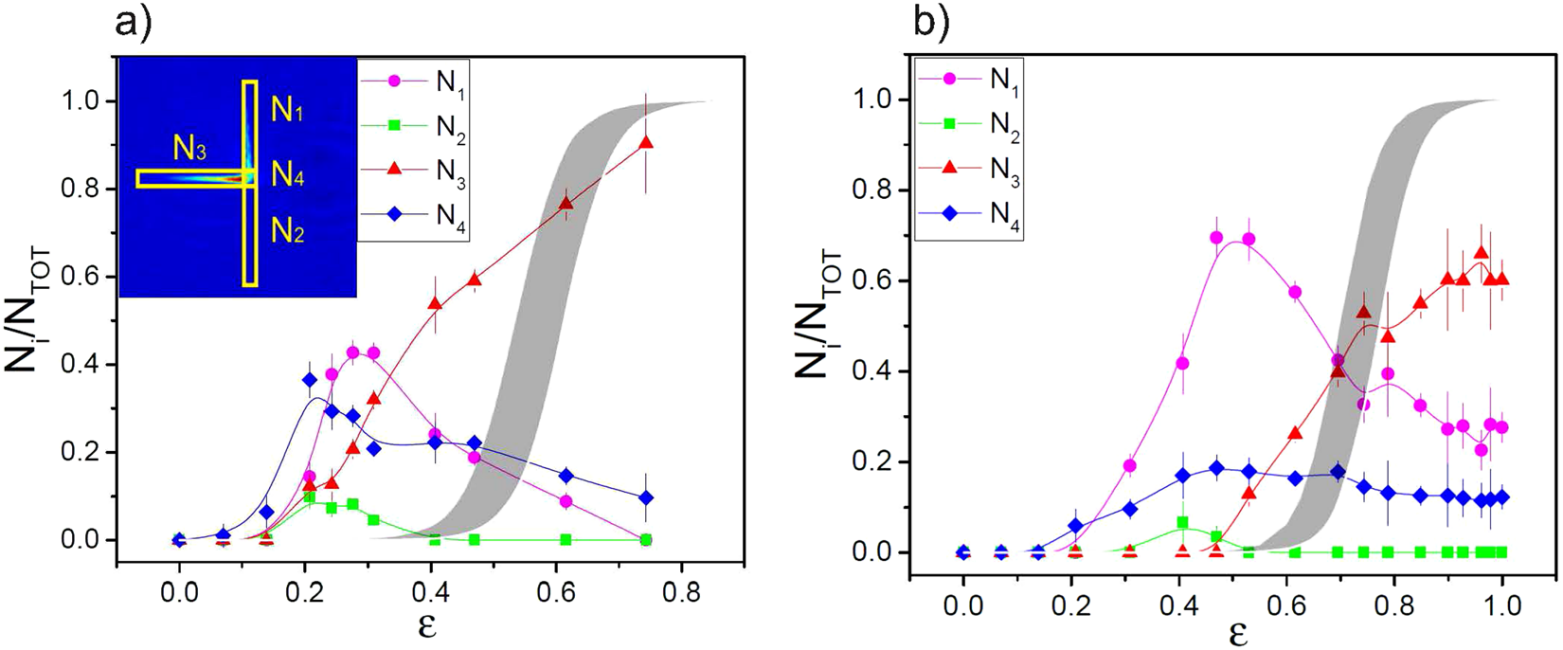
Relative population of the different classes of non-transmitted atoms (inset) as a function of the lattice height, revealing the complexity and different mechanisms governing the atom dynamics in the splitter. Measurements are shown for two different regimes: (**a**) v = 2.7v_*R*_ and (**b**) v = 3.7v_*R*_. Solid grey areas show the maximum quantum reflection probability by a single barrier calculated using the analytical expression in ref. 17. Solid lines are guides to the eyes. Please refer to the insets of Fig. 3 for the definition of the waveguides’ orientation.

A complete analysis of the way in which the atoms redistribute among the different channels of the distributed quasi-Bragg splitter, as a function of the lattice height, helps to highlight the mechanisms which determine the dynamics in the different velocity regimes. Figure 8 shows the behaviour of the non-transmitted atoms in a regime of intermediate and high velocity. We observe in particular a reduction of the atoms trapped in the centre (*N*_4_) or reflected into the second channel of WG2 (*N*_2_). A different onset of the blockage of the atoms before the crossing region is also observed (*N*_1_), likely following the onset of the quantum reflection from the single barriers of the inhomogeneous lattice.
